# Therapeutic Drug Monitoring of Orally Administered Letermovir Prophylaxis in Allogeneic Hematopoietic Stem Cell Transplant Recipients

**DOI:** 10.1128/aac.00657-22

**Published:** 2022-07-25

**Authors:** Léna Royston, Stavroula Masouridi-Levrat, Verena Gotta, Eva Royston, Caroline Pressacco-Brossier, Yasmine Abi Aad, David Tonoli, Abderrahim Karmime, Murielle Jayo, Christian Van Delden, Pierre Lescuyer, Marc Pfister, Yves Chalandon, Dionysios Neofytos

**Affiliations:** a Division of Infectious Diseases, University Hospital of Geneva, Geneva, Switzerland; b Division of Hematology, Bone Marrow Transplant Unit, University Hospital of Geneva and Faculty of Medicine, University of Geneva, Geneva, Switzerland; c Division of Pediatric Pharmacology and Pharmacometrics, University of Basel Children's Hospital, Basel, Switzerland; d Division of Laboratory Medicine, University Hospital of Geneva, Geneva, Switzerland

**Keywords:** letermovir, therapeutic drug monitoring, CMV, prophylaxis, allogeneic hematopoietic stem cell transplant recipients, cytomegalovirus

## Abstract

With balanced safety-efficacy profile, letermovir anti-cytomegalovirus (CMV) prophylaxis is used in hematopoietic stem cell transplant recipients (HSCTR). We assessed feasibility and usefulness of letermovir therapeutic drug monitoring (TDM) in HSCTR. We performed a prospective observational study on letermovir-TDM including 40 consecutive adult CMV-seropositive allogeneic-HSCTR who received orally (PO) administered letermovir. Minimal blood concentrations of letermovir (C_trough_) were measured on days 3 and 7 postletermovir initiation and weekly thereafter. Letermovir-C_trough_ remained stable during the first 70 days post-HSCT at a median of 286 μg/L (interquartile range, 131 to 591 μg/L), with large interpatient/intrapatient variability. No associations between breakthrough clinically significant CMV infection or detectable CMV DNAemia and letermovir-C_trough_ were observed. Patients with letermovir-associated adverse events had higher letermovir-C_trough_ than patients without (400 versus 266 μg/L, *P *= 0.02). Letermovir-C_trough_ was similar in patients with or without gastrointestinal symptoms (280 versus 300 μg/L, *P = *0.49). Acute grade ≥2 GvHD was associated with higher letermovir-C_trough_ (479 versus 248 μg/L, *P *= 0.001), including gastrointestinal GvHD (499 versus 263 μg/L, *P *= 0.004). Concomitantly administered posaconazole and cyclosporine were associated with higher letermovir-C_trough_ (707 versus 259 μg/L, *P *< 0.001 and 437 versus 248 μg/L, *P *= 0.01, respectively). In multivariable analysis, both posaconazole (odds ratio [OR], 4.9; 95% confidence interval [CI], 2.4 to 9.7; *P *< 0.0001) and cyclosporine-adjusted letermovir dose at 240 mg daily (OR, 3.5; 95% CI, 1.4 to 9.0; *P *= 0.01) were independently associated with higher letermovir-C_trough_. In conclusion, administration of PO letermovir led to measurable and relatively stable letermovir-C_trough_, without noticeable associations with clinical efficacy. Letermovir exposure was not affected by gastrointestinal symptoms, but with posaconazole and cyclosporine administration. Associations between letermovir and concomitantly administered agents and adverse events warrant additional clinical studies.

## INTRODUCTION

Cytomegalovirus (CMV) infection is the most frequent infectious disease complication postallogeneic hematopoietic stem cell transplant (HSCT), with significant direct and indirect clinical consequences and associated costs (1–6). Letermovir is an antiviral agent recently approved for primary CMV prophylaxis during the first 14 weeks after allogeneic HSCT (7). Its efficacy and safety have been demonstrated in a prospective randomized placebo-control clinical trial (8). However, even in this pivotal clinical trial, more than one-third of patients on letermovir prophylaxis developed a breakthrough clinically significant (cs) CMV infection and already one patient exhibited letermovir resistance (8). Although one-third of patients received intravenously (IV) administered letermovir, most patients received letermovir orally (PO), which is relevant as allogeneic HSCT recipients (R) frequently develop high-degree gastrointestinal (GI) mucositis and/or graft-versus-host disease (GvHD), both associated with suboptimal drug absorption (9, 10).

In May 2019, administration of letermovir-based primary CMV-prophylaxis was initiated in high-risk allogeneic HSCTR at our institution with a breakthrough csCMV rate of 27% (11, 12). Due to nationwide shortage in IV letermovir, only PO letermovir was used. We hypothesized that allogeneic HSCTR with GI mucositis or GvHD may have suboptimal absorption of PO letermovir, potentially associated with higher rates of breakthrough csCMV infection. We performed a prospective observational study to assess the trough blood concentrations of PO letermovir in allogeneic HSCTR by performing prospective letermovir therapeutic drug monitoring (TDM).

## RESULTS

### Patient population.

Forty consecutive adult allogeneic HSCTR were included and followed for a mean of 65 days (interquartile range [IQR], 42, 72; range, 1, 76) (Table 1). Median age was 57 years (IQR, 44.3, 68.8; range, 22, 77) and 14 patients (35%) were female. Indication for letermovir was primary prophylaxis post-HSCT for 33 patients (83%) and prophylaxis during greater than or equal to grade 2 acute GvHD for 7 (18%) patients. Nineteen patients (48%) developed greater than or equal to grade 2 acute GvHD during follow-up, at a median of 23 days post-HSCT (IQR, 20, 46; range, 12, 267). Thirty-five patients (88%) received 480 mg/day of letermovir, four patients (10%) received 240 mg/day, and one patient started with 480 mg/day and was switched to 240 mg/day during follow-up due to cyclosporine initiation. Letermovir was continued until day 70 in 24 (60%) patients. In eight patients (20%), letermovir was discontinued at a median of 25 days (range, 5, 54) for csCMV infection. Letermovir was interrupted in four patients (10%) at a median of 24 days (range, 18, 42) due to HHV-6 reactivation and was restarted in three of those patients at a median of 61 days.

**TABLE 1:** Baseline patient characteristics^*a*^

Patient and HCT characteristics	Patients, *n* = 40 (%)
Demographics	
Age (yr), mean (SD, range)	55 (14.9, 34–74)
Gender, female	14 (35)
BMI	25 (4.6, 17.6–36.4)
Underlying disease	
Acute myeloid leukemia	22 (55)
Myelodysplasic syndrome	3 (8)
Acute lymphoblastic leukemia	5 (13)
Lymphoma	5 (13)
Other^*b*^	5 (13)
HSCT characteristics	
Conditioning, MAC	9 (23)
HSCT donor	
HLA-matched related	6 (15)
HLA-matched unrelated	18 (45)
HLA-mismatched unrelated	2 (5)
Haploidentical	14 (35)
HSCT source	
Bone marrow	1 (3)
Peripheral blood	39 (98)
Engraftment day, mean (SD, range)	19 (4.6, 7–32)
GvHD grade ≥2	
aGvHD during follow-up	19 (48)
aGvHD at baseline	7 (18)
Day post-HSCT, mean (SD, range)	49 (62.6, 12–267)
Chronic GvHD	1 (2)
Day post-HSCT, mean (SD, range)	140
CMV serological status	
Donor+/recipient+	21 (53)
Donor–/recipient+	19 (47)

a BMI, body mass index; SD, standard deviation; HSCT, hematopoietic stem cell transplant; MAC, myeloablative conditioning; HLA, human leukocyte antigen; aGvHD, acute graft versus host disease; CMV, cytomegalovirus.

b Includes 1 patient with chronic lymphoblastic leukemia, 1 patient with biphenotypic acute leukemia, 2 patients with multiple myeloma, and 1 patient with sickle cell disease.

### Letermovir TDM.

**FIG 1:**
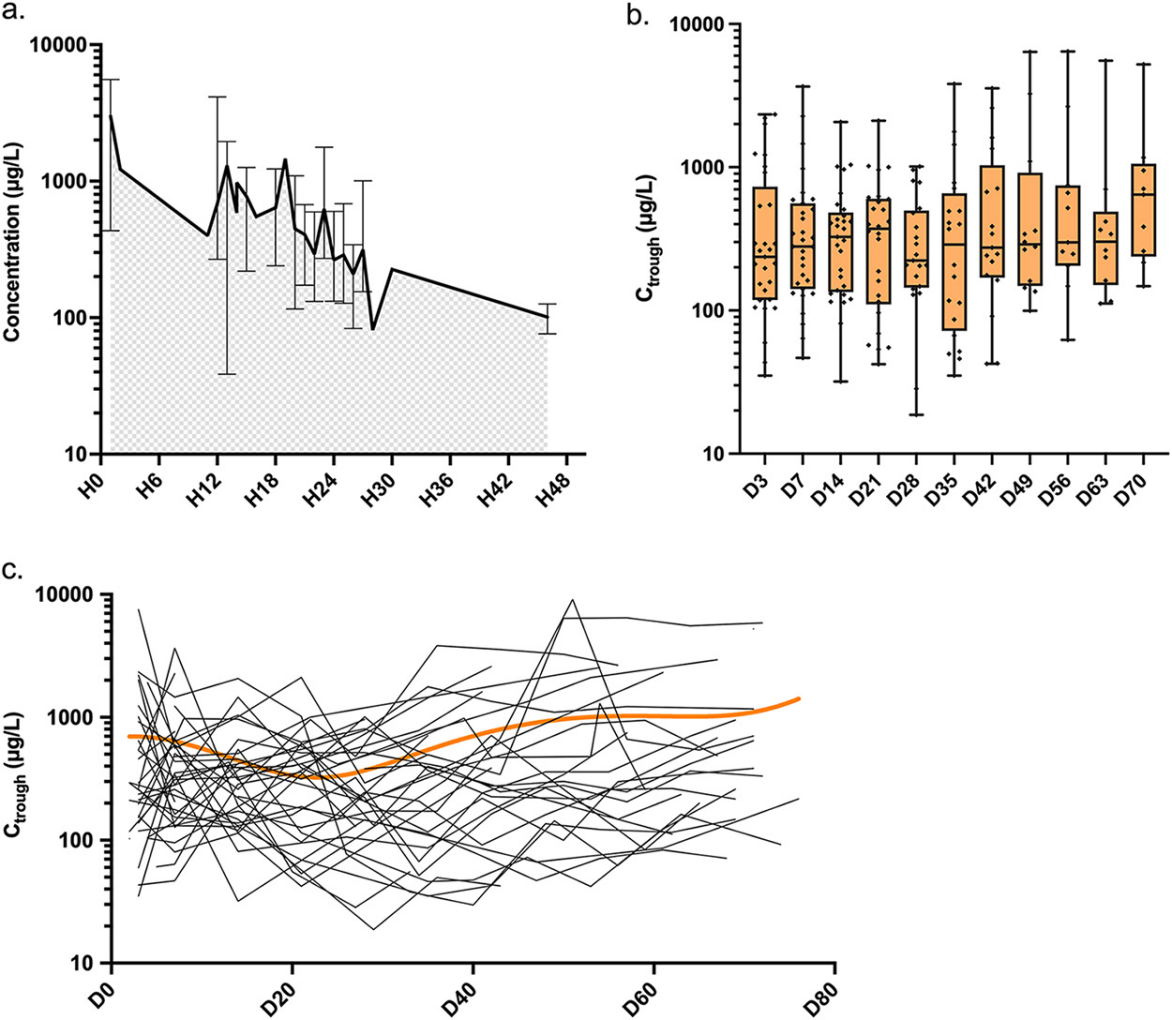
Distribution of 296 letermovir plasma trough concentrations (C_trough_) in 40 hematopoietic stem cell transplant recipients. (a) Letermovir plasma concentrations in a log scale, based on the timing of measurement after the last letermovir administered dose. Although all efforts were made to obtain C_trough_ measurements only, 33 of 296 (11.2%) measurements were not real trough levels. (b) Weekly average letermovir-C_trough_ during the 70-day follow-up period, presented as boxplots with whiskers representing minimum/maximum values, in a log scale. (c) Daily C_trough_ presented as individual values in a log scale, and nonlinear regression (in red) at the fifth polynomial degree. C_trough_, letermovir trough concentration; H, hours postletermovir administration; D, days postletermovir administration.

In total, 296 TDM values were collected, corresponding to a median of 8 (range, 1, 11) TDM values per patient. Thirty-three samples (11.2%) were not real trough levels. Letermovir concentrations peaked after administration of the drug and rapidly declined to reach a plateau (Figure 1a). A total of 263/296 (88.8%) values corresponded to letermovir trough concentrations (C_trough_) (median, 7 values per patient; range, 0, 11), with a mean and median concentration of 637 and 286 μg/L (range, 18.7, 9,089 μg/L), respectively. The distribution of letermovir-C_trough_ mean, median, standard deviation, IQR, and range are detailed in Table 2. No significant variability was observed across the different time points (*P = *0.85, Figure 1b and c). Interindividual and residual intraindividual variabilities were estimated to be 84% and 114%, respectively (Figure 1c).

**TABLE 2:** Measured letermovir trough concentrations^*a*^

Clinical circumstances	Mean	SD	Range	Median	IQR
CMV					
csCMV	466	510	103–1,408	205	129–947
No csCMV	781	1023	100–5,230	487	246–833
CMV >21 IU/mL	579	637	55–2,540	409	118–598
CMV >100 IU/mL	578	661	55–2,540	386	112–595
CMV >150 IU/mL	604	834	55–2,540	319	101–847
LET-associated adverse events					
Presence of adverse events	1,311	2,006	43–6,437	400	206–1,220
No adverse events	532	866	19–9,089	266	125–554
Acute GvHD grade ≥2					
Presence of aGvHD	1,297	1,725	35–6,437	479	311–1,265
Presence of GI aGvHD	1,334	2,018	35–6,437	499	272–1,003
Presence of non-GI aGvHD	1,791	2,020	87–6,537	678	342–2,940
No aGvHD	488	851	19–9,089	248	121–536
Concurrent medication					
Posaconazole	1,495	1,786	52–7,520	758	326–2,108
No posaconazole	464	810	19–9,089	259	122–503
Corticosteroids	1,162	1,504	35–6,437	507	287–1,280
No corticosteroids	404	789	19–9,089	215	117–418
Cyclosporin	1,123	875	259–2,940	980	455–1,720
No cyclosporine	664	1,257	19–9,089	259	119–542

a Data are in μg/L. SD, standard deviation; IQR, interquartile range; LET, letermovir; aGvHD, acute graft versus host disease; csCMV, clinically significant cytomegalovirus infection.

### Letermovir efficacy.

**FIG 2:**
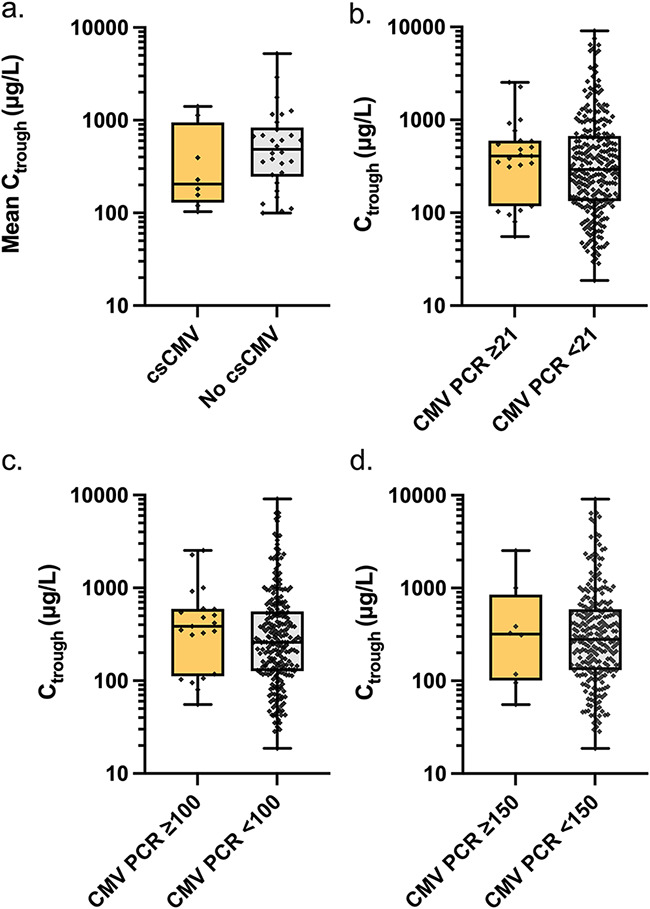
Associations between letermovir plasma trough concentration (C_trough_) and CMV DNAemia. (a) Mean letermovir-C_trough_ throughout the study in patients who developed a breakthrough clinically significant (cs) CMV (necessitating preemptive anti-CMV treatment initiation) compared to patients who did not develop breakthrough csCMV. (b to d) Letermovir-C_trough_ at the time of CMV DNAemia >21 IU/mL (level of detectability) (b), >100 IU/mL (c), and > 150 IU/mL (d). There were no statistically significant differences between the groups compared. csCMV, clinically significant CMV infection; C_trough_, letermovir trough concentration. Data are represented as boxplots in a log scale, with whiskers representing minimum and maximum values.

The cumulative incidence of breakthrough csCMV infection during follow-up was 22.5% (9/40): eight patients and one patient, when using the >150 and >500 IU/mL threshold, respectively. Mean letermovir-C_trough_ throughout the study did not significantly differ between patients with and without breakthrough csCMV (*P = *0.24; Figure 2a). Letermovir-C_trough_ did not significantly differ at the time of CMV DNAemia above and below the following thresholds: 21 IU/mL (detection threshold), 100 IU/mL, or 150 IU/mL (*P = *0.44, 0.30, and 0.95, respectively; Figure 2b to d). In univariable analyses there were no associations between CMV DNAemia >150 IU/mL (odds ratio [OR], 0.99; 95% confidence interval [CI], 0.99, 1.00; *P *= 0.93) or csCMV infection (OR, 0.99; 95% CI, 0.99, 1.00; *P *= 0.15) and letermovir-C_trough_.

### Letermovir safety.

**FIG 3:**
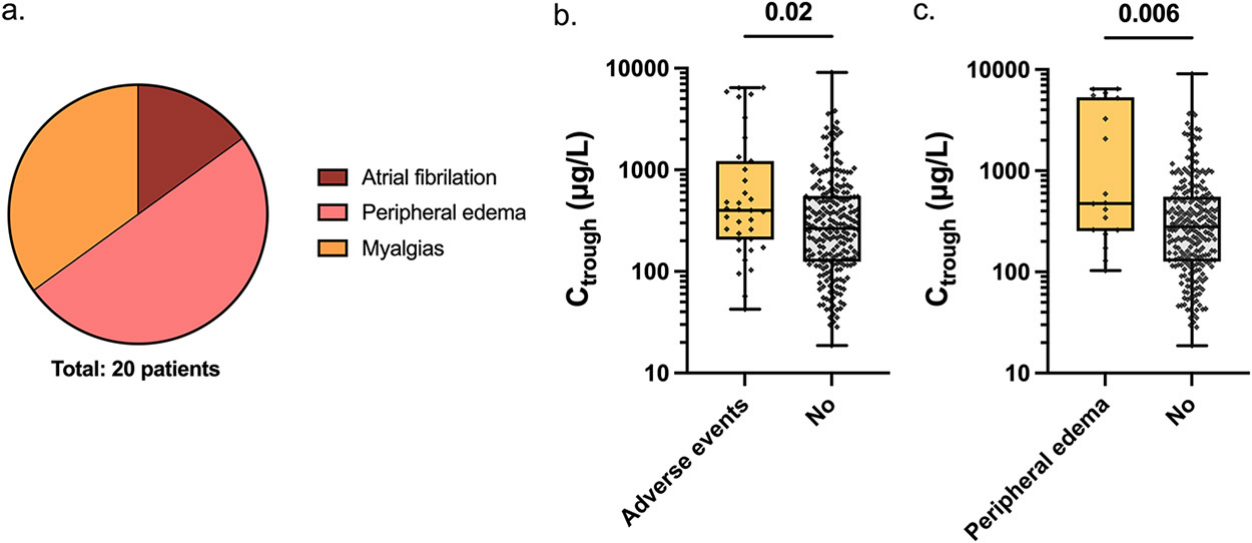
Associations between letermovir plasma trough concentration and adverse events, including atrial fibrillation, peripheral edema, and myalgias. (a) Number of patients who developed a potentially letermovir-associated adverse event during follow-up. (b) Letermovir trough concentrations at the time of a potential adverse event. (c) Letermovir trough concentrations at the time of peripheral edema diagnosis. C_trough_, letermovir trough concentration. Data are represented as boxplots in a log scale, with whiskers representing minimum and maximum values. Significant *P* values (<0.05) are presented.

Relevant laboratory and clinical variables were assessed as potential letermovir-associated adverse events. There were no associations between letermovir-C_trough_ and renal or liver function tests (Fig. S1). The following prospectively monitored clinical variables were observed (Figure 3a): atrial fibrillation (3, 8%), peripheral edema (10, 25%), and myalgias (7, 18%). The median letermovir-C_trough_ was significantly higher in patients with any of the above adverse events (median, 400 μg/L) when compared to patients without (median, 266 μg/L; *P = *0.02; Figure 3b). In particular, the 10 patients with peripheral edema had significantly higher median letermovir-C_trough_ (median, 476 μg/L) compared to patients without (median, 279 μg/L; *P *= 0.006; Figure 3c). Univariable analyses demonstrated a significant association between adverse events (AEs) and letermovir-C_trough_ (OR, 1.00; 95% CI, 1.00, 1.00; *P *= 0.007). Due to small numbers of events, separate analyses for each one of the AEs observed were not performed.

### Letermovir TDM, GI symptoms, and GvHD.

**FIG 4:**
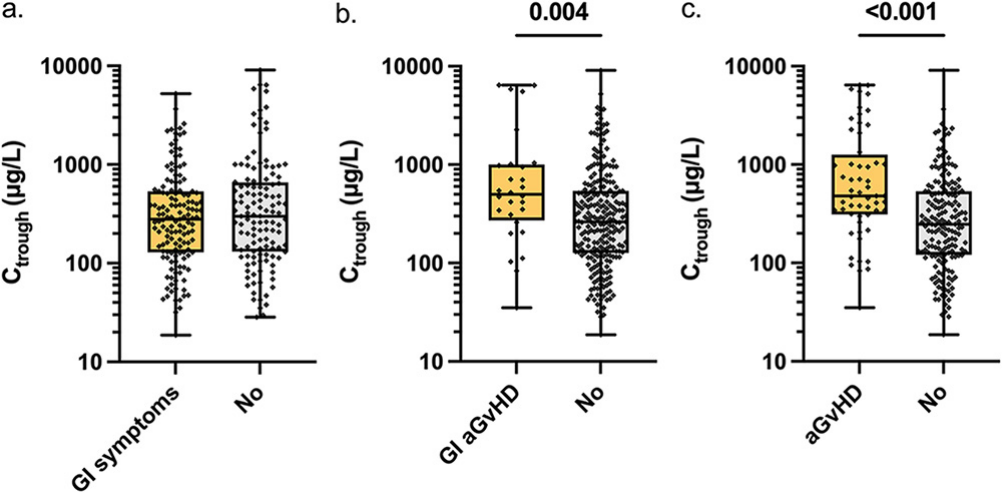
Associations between letermovir plasma trough concentration, gastrointestinal (GI) symptoms and acute graft versus host disease (aGvHD). Letermovir trough concentrations in patients with and without any GI symptoms in general, including nausea, vomiting, or diarrhea (a), greater of equal to grade 2 acute GI GvHD (b), and greater or equal to grade 2 acute GvHD (c). C_trough_, letermovir trough concentration; GI, gastrointestinal; aGvHD, acute graft-versus-host disease. Data are represented as boxplots in a log scale, with whiskers representing minimum and maximum values. Significant *P* values (<0.05) are presented.

Letermovir-C_trough_ did not significantly differ based on the presence (median, 280 μg/L) or not (median, 300 μg/L) of GI symptoms (nausea, vomiting, diarrhea; *P = *0.49; Figure 4a). In addition, there was no difference in letermovir-C_trough_ based on diarrhea severity: no diarrhea versus one to three diarrhea episodes per day versus more than three diarrhea episodes per day (median, 289 versus 310 versus 283 μg/L, respectively; *P = *0.93). In contrast, in patients with GI GvHD, letermovir concentrations were significantly higher (median, 499 μg/L) when compared to patients without GI GvHD (263 μg/L; *P = *0.004; Figure 4b). Similarly, letermovir-C_trough_ were higher during concurrent acute GvHD diagnosis affecting any organ (median, 479 μg/L) versus not (median, 248 μg/L; *P = *0.001; Figure 4c).

### Letermovir TDM and concomitant drugs.

**FIG 5:**
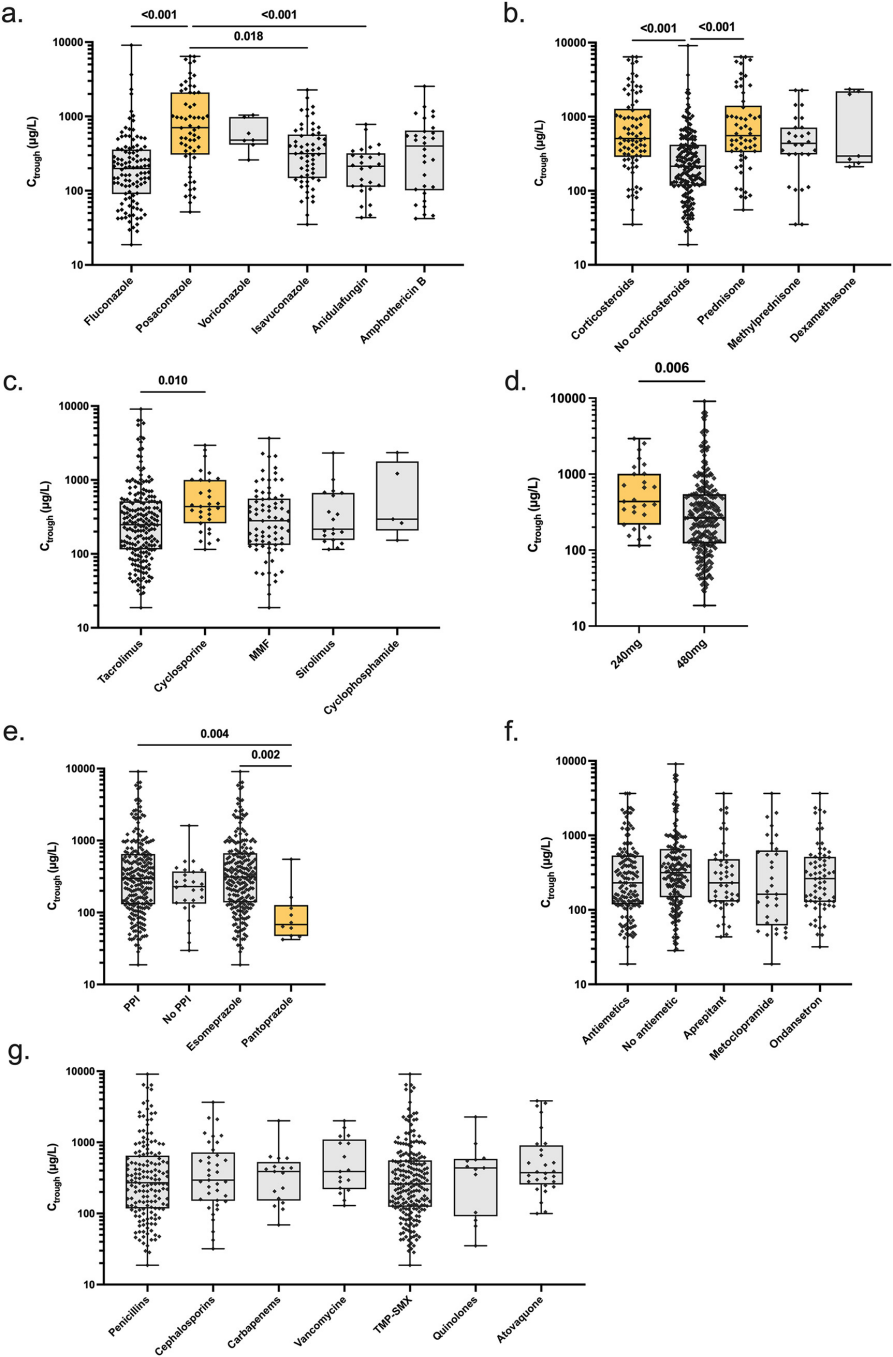
Associations between letermovir plasma trough concentration and other concomitantly administered medications, including antifungal agents (a), corticosteroids (b), immunosuppressive agents (c), letermovir administered dose (d), proton-pump inhibitors (e), antiemetics (f), and antibacterial agents (g). For panels a, c, d, and g, all patients were receiving at least one drug from the respective class. Data are represented as boxplots in a log scale, with whiskers representing minimum and maximum values. Significant *P* values (<0.05) are presented. MMF, mycophenolate mofetil; PPI, proton-pump inhibitors; TMP-SMX, cotrimoxazole.

Letermovir-C_trough_ levels were studied based on concomitant administration of different drugs. Among antifungal drugs received as prophylaxis or treatment, concomitant posaconazole administration was associated with higher letermovir-C_trough_ (median, 707 μg/L) compared to fluconazole, isavuconazole, or anidulafungin (*P *< 0.001, *P *= 0.02, and *P *< 0.001, respectively; Figure 5a). Concomitant administration of systemic corticosteroids was associated with higher letermovir-C_trough_ (median, 507 versus 215 μg/L; *P *< 0.001; Figure 5b). Notably, prednisone was associated with increased C_trough_ (median, 555 versus 215 μg/L; *P *< 0.001). Among immunosuppressive agents, cyclosporine was associated with increased C_trough_ compared to tacrolimus (median, 437 versus 248 μg/L; *P = *0.01; Figure 5c), which is concordant with higher letermovir-C_trough_ in patients receiving 240 mg/day of letermovir versus 480 mg/day (median, 437 versus 265 μg/L, respectively; *P = *0.006; Figure 5d). Pantoprazole administration was associated with decreased C_trough_ compared to esomeprazole (median, 69 versus 311 μg/L; *P = *0.002; Figure 5e). In contrast, concomitant administration of different antiemetics or different classes of antibacterial agents did not have any effect on letermovir-C_trough_ (Figure 5f and g).

### Risk factors for high letermovir-C_trough_.

**FIG 6:**
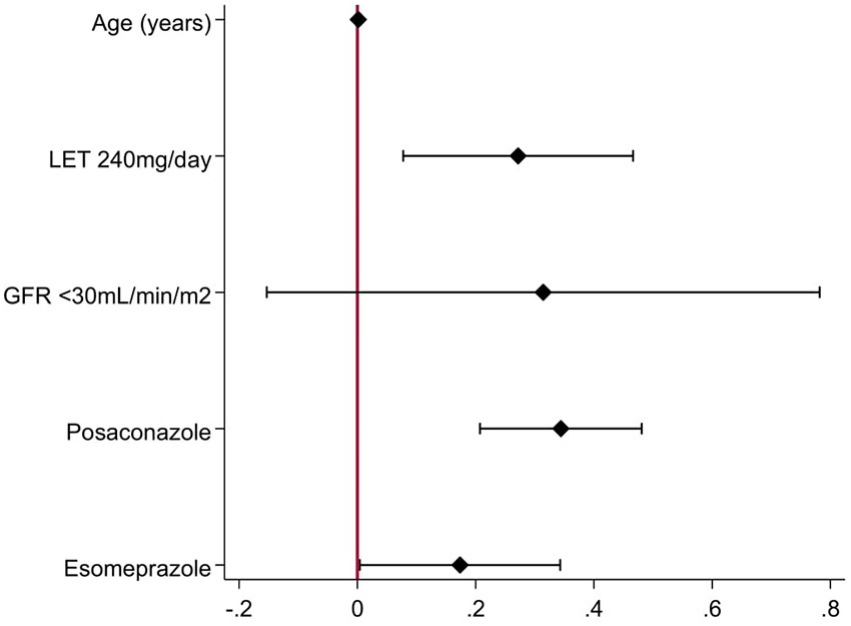
Forest plot of adjusted odds ratio and confidence intervals of the multivariable logistic regression model regarding predictors of letermovir-C_trough_ above the median value of 286 μg/L. LET, letermovir; GFR, glomerular filtration rate, in mL/min/1.73 m^2^.

Univariable analyses were performed using demographics, HCT-related variables, underlying renal and liver function, and coadministered agents, to identify risk factors for high letermovir-C_trough_. As a letermovir-C_trough_ cutoff has not, as yet, been defined, we considered the median letermovir-C_trough_ (286 μg/L) as the studied outcome. All clinically relevant variables with a *P *< 0.10 in univariable analyses were entered in a multivariable logistic regression model in a stepwise fashion, after excluding those with important interactions (data not shown). Due to significant interactions between aGvHD, corticosteroids, and posaconazole, the former were not included in the final model (Fig. 6). Coadministration of posaconazole (OR, 4.9; 95% CI, 2.4; 9.7; *P* < 0.0001) and cyclosporine-adjusted letermovir dose at 240 mg once daily (OR, 3.5; 95% CI, 1.4; 9.0; *P *= 0.01) was significantly associated with higher than median letermovir-C_trough._ A trend for higher letermovir trough concentrations and coadministration with esomeprazole was also identified (OR, 2.2; 95% CI, 0.2; 3.4; *P* = 0.06).

## DISCUSSION

Although letermovir is widely used for CMV prophylaxis in allogeneic HSCT recipients, its pharmacokinetic properties in real life remain poorly defined. In this first prospective letermovir TDM study, we aimed to describe letermovir-C_trough_ and explore the determinants of letermovir plasma concentrations in a real-world setting. We observed a median letermovir-C_trough_ of 286 μg/L, which is consistent with previously reported data in healthy volunteers who received ascending doses of letermovir with a reported C_trough_ of 193 μg/L (13). In contrast, median untimed letermovir concentrations in 26 patients of 2,246 μg/L, ranging from not detectable to 24,250 μg/L, have been reported in the organ transplant literature (14). It is likely that the use of untimed sample collection instead of C_trough_, as well as differences in the testing method, might have contributed to this large variability in reported concentrations. Furthermore, intraindividual changes may, in part, explain those discrepancies, as suggested by the large intraindividual and interindividual variability among our patients. However, our data are consistent with data reported in Phase 1 clinical trials for the validation of letermovir in healthy volunteers and suggest that allogeneic HSCT recipients, despite multiple comorbidities and significant polypharmacy, appear to have similar plasma trough concentrations. Notably, our data suggest that letermovir concentrations remain relatively stable during the first 70 days after an allogeneic HSCT, without any significant differences observed between day 3 and day 70.

We did not identify any significant association between breakthrough csCMV infection and letermovir plasma concentrations. This finding is consistent with a recent report from Prohn et al. (10) showing the absence of letermovir exposure dependencies for csCMV infection at week 14 or week 24 post-HSCT. Notably, the threshold for CMV preemptive treatment and csCMV infection definition at our institution changed from >150 to >500 IU/mL during the study period. Hence, our observations with regard to breakthrough csCMV infection and letermovir TDM are nonconclusive. However, when we looked at CMV DNAemia at different thresholds (>21, 100, and 500 IU/mL), there was no significant association between any of the above with letermovir-C_trough_ values. Similarly, univariable analyses failed to identify any potential associations between csCMV infection and letermovir-C_trough_. In contrast, potential adverse events (atrial fibrillation, peripheral edema, myalgias) were associated with higher letermovir-C_trough_. Although causality has not been shown previously and could not be attributed in this observational study, the above may suggest that although the current dosing recommendations allow for sufficient letermovir exposure to avoid csCMV infections, higher letermovir-C_trough_ may require further monitoring to prevent from letermovir-related adverse events. This observation needs to be further investigated in the future.

Gastrointestinal symptoms, including nausea, vomiting, and diarrhea (even severe diarrhea, at >3 episodes per day), did not appear to have an effect on letermovir-C_trough_. This is pertinent information, considering that all patients in this study received PO letermovir. Therefore, based on our data and the existing body of evidence, PO letermovir appears to be well absorbed and associated with detectable C_trough_ values even in patients with impaired GI tract function. Whether administration of IV letermovir in those high-risk patients could potentially lead to higher plasma C_trough_ and improved clinical outcomes remains to be further investigated. Of note, letermovir-C_trough_ was significantly higher in patients with moderate to severe GI tract GvHD when compared to patients without GvHD. Similarly, patients with non-GI tract GvHD had higher C_trough_ compared to patients without GvHD. The higher concentrations observed in patients with GI GvHD suggest that PO letermovir remains an option even in patients with severe gastrointestinal symptoms, likely due to potential associations with GvHD concomitant treatments.

Indeed, higher letermovir concentrations were observed in patients receiving posaconazole and corticosteroids. This may, in part, account for the higher letermovir-C_trough_ observed in patients with aGvHD, as treatment with corticosteroids and primary antifungal prophylaxis with posaconazole is routinely administered in those patients per institutional protocol and international guidelines (15). This potential interaction has not yet been described. Letermovir is partly metabolized by glucuronidation, through uridine 5′-diphospho-glucuronosyltransferase 1A1/1A3 and is a substrate of OATP1B1/3 hepatic transporters (16). In addition, letermovir is a moderate cytochrome P450 (CYP) 3A4/5 and CYP2C8 inhibitor and an OATP1B1/3 and P-glycoprotein inhibitor and may induce CYP2C19 and CYP2C9 (7, 17). Recent data suggest that letermovir coadministration in healthy subjects and HSCT recipients did not induced significant alterations on posaconazole, isavuconazole, or fluconazole concentrations (18–20). However, the inverse effect of antifungal agents on letermovir concentrations remains unknown. Our data suggest that coadministration of posaconazole may be associated with higher letermovir concentrations. We hypothesized that a potential interaction between posaconazole and letermovir could be due to both agents using the P-glycoprotein metabolism pathway. This could also potentially explain that no association was identified between other antifungal agents, notably voriconazole and isavuconazole, and letermovir concentrations. Although the latter may, in part, be attributed to the low numbers of patients treated with those agents making further conclusions difficult to make, it may also suggest absence of further interactions via the CYP3A4 pathway, common for all those three azoles. Further dedicated pharmacological studies will be required to understand the mechanistic link between letermovir and these drugs.

Concomitant administration of various antibacterial agents or antiemetics did not have any effect on plasma letermovir concentrations. In contrast and as previously described, higher letermovir-C_trough_ were found in patients receiving GvHD prophylaxis with cyclosporine compared to tacrolimus, despite the letermovir dose reduction in this context due to previously described drug-drug interactions (16, 21). Among proton-pump inhibitors, no difference could be observed with or without those agents; however, patients receiving pantoprazole appeared to have lower letermovir levels than those receiving esomeprazole. Due to low numbers of patients receiving those agents, no further conclusions could be drawn.

In conclusion, our study describes the pharmacokinetic profile of orally administered letermovir in a real-world cohort of high-risk allogeneic HSCT recipients. This study is limited by its small patient size and its observational nature, which prevented the identification of underlying mechanisms responsible for modifications of letermovir-C_trough_. Follow-up was limited for logistical reasons to the first 70 days posttransplant, not extending through day 100, the usual duration of letermovir prophylaxis administration. In addition, as the threshold for preemptive CMV treatment was changed during the study period, our observations do not allow for definitive conclusions with regard to potential associations between letermovir-C_trough_ and breakthrough csCMV infection. However, clinically relevant observations could still be found. Our findings indicate that PO administered letermovir is well absorbed and C_trough_ are not significantly affected by GI tract symptomatology. In fact, GvHD, including severe GI GvHD, appeared to be associated with higher letermovir concentrations, most likely due to coadministration of posaconazole as primary antifungal prophylaxis in the setting. While letermovir TDM does not appear to be required to ascertain clinical efficacy, our preliminary findings suggest a potential application in clinical practice, to further mitigate the risk of experiencing letermovir-induced safety events. Additional clinical studies are warranted to investigate the potential associations of letermovir with concomitantly administered posaconazole and the role of TDM in personalizing dosing to further optimize the safety-efficacy balance of letermovir in HSCT recipients and ultimately improve posttransplant clinical outcomes.

## MATERIALS AND METHODS

### Study design.

This was a prospective noninterventional open-label study. All consecutive adult (≥18-year-old) CMV-seropositive allogeneic HSCTR who received primary CMV-prophylaxis with PO letermovir between March 1, 2020 and April, 20, 2021 were included. Letermovir was administered at 480 mg once daily or 240 mg once daily in case of cyclosporine coadministration (22). The study was approved by the local Ethics Committee, and all patients signed an informed consent form before participating in this study.

### Study outcomes.

The primary objective was to describe the minimal blood concentrations (trough concentration [C_trough_]) of PO letermovir in a cohort of allogeneic HSCTR. The following secondary objectives were assessed: (i) efficacy and safety of PO letermovir prophylaxis and (ii) distribution of letermovir TDM during mucositis, GI and other grade ≥2 GvHD, and based on concomitantly administered agents.

### Study procedures.

Letermovir-C_trough_ was measured on day 3 (±1), day 7 (±1) postletermovir prophylaxis initiation, and weekly (±1 day) thereafter, for a total of maximum 11 samples per patient. Measurement of letermovir plasma concentration was performed by the institutional Toxicology and TDM Laboratory using an in-house developed ultra-high performance liquid chromatography tandem mass spectrometry assay. The method was validated according to the Clinical Laboratory Standards Insitute guidelines, considering: linearity, precision and accuracy on inter- and intrabatch series, lower limit of quantification (LLOQ), dilution integrity, carryover, matrix effects, interferences (hemolyzed, lipemic and icteric plasma, as well as selected drugs), and stability (preanalytical and analytical: freeze/thaw cycles stability, benchtop stability, processed sample stability, long-term stability, and stock solution stability). The method was found to be linear from 1 to 2,500 μg/L. LLOQ was validated at 1 μg/L. Solely total letermovir concentrations were measured and reported for this assay. Pertinent baseline HSCT-associated variables, including conditioning regimen and HSCT-type were collected. The following variables were routinely collected prospectively for the first 10 weeks of letermovir administration: HSCT-associated complications (e.g., GvHD, mucositis), GI symptoms (nausea, vomiting, diarrhea, including diarrhea frequency), concomitantly administered drugs, plasma CMV quantitative PCR (qPCR) and letermovir TDM data, renal and hepatic function, and selected symptoms potentially associated with letermovir administration (new-onset atrial fibrillation, peripheral lower extremity edema, and myalgias) (8).

### Institutional practices.

Monitoring and preemptive treatment of CMV DNAemia at our institution have been previously described (11). Briefly, plasma CMV qPCR is performed once weekly at our institution in allogeneic HSCTR during the first 3 months post-HSCT with the COBAS CMV for Cobas 6800 test (Roche Diagnostics, Indianopolis, IN, USA) with a limit of detection of 21 IU/mL and limit of quantification of 25 IU/mL. Until December 31, 2020, primary letermovir CMV-prophylaxis was administered to (i) all CMV donor-negative (D−)/R-positive (R+) patients from day (D) 1 to D100 post-HSCT and (ii) CMV HSCTR+ with early (during the first 6 months post-HSCT) grade ≥2 acute GvHD requiring corticosteroid treatment at ≥1 mg/kg/day and until tapering to <10 mg/day of prednisone equivalent (11). Starting January 1, 2021, all CMV HSCTR+ received primary CMV prophylaxis with letermovir between days 1 and 100 post-HSCT. Until December 31, 2020, csCMV infection prompting CMV-preemptive treatment initiation was defined based on consensus international guidelines adjusted to our institutional practices using a CMV DNAemia cutoff >150 IU/mL (23, 24). During the study period, new evidence suggested that low-grade CMV DNAemia in patients treated with letermovir may represent aborted viral replication rather than effective viral replication (25). Therefore, the CMV DNAemia threshold for csCMV infection and CMV preemptive treatment initiation was changed from >150 to >500 IU/mL as of January 1, 2021.

### Definitions.

Clinically significant CMV infection was defined as detailed above. Study inclusion day was the date of letermovir initiation and patients were followed for the first 70 days after study inclusion or until the end of letermovir administration, if stopped before day 70. The 70-day follow-up was chosen for logistical/feasibility reasons, considering the weekly follow-up of our patients during that period. Letermovir-C_trough_ was defined as a sample drawn 24 (±2) hours after last letermovir administration and before the administration of the next dose.

### Statistical analysis.

Standard descriptive statistics were used to summarize the study population characteristics. The Fisher’s exact or chi-square tests were used for categorical variables and two-tailed Student *t* test for continuous variables. Continuous variables are presented as means with standard deviation and range, or as medians with IQR, as appropriated. The letermovir TDM values were presented as medians at each time point measured with range and interquartile range. The overall mean letermovir-C_trough_ was calculated on log-transformed data, accounting for repeated measurements (linear mixed effect model). Estimates of interindividual and residual intraindividual variability were derived in terms of percent coefficient of variation. Differences in TDM among groups were identified with Mann-Whitney or Kruskal-Wallis tests, as appropriate. Two-sided tests were performed, and a *P *< 0.05 was considered as statistically significant. Univariable analyses were performed to identify potential associations between letermovir-C_trough_ and clinical efficacy (e.g., CMV DNAemia, csCMV infection) and toxicity (AEs: atrial fibrillation, myalgias, and peripheral edema). Univariable analyses were performed to identify predictors of high letermovir-C_trough_, with the following independent variables studied: demographics, HCT characteristics, laboratory values reflecting bone marrow, renal and liver function, and coadministered medications. Variables with a *P *< 0.10 in univariable analyses and after considering potential interactions among them using the Pearce correlation test were entered in a stepwise fashion into a multivariable model. Results are presented as OR with 95% CI. Data were analyzed using STATA 14 statistical software (StataCorp, College Station, TX, USA) and GraphPad Prism 9 (GraphPad Software Inc., San Diego, CA, USA).
