# Supplementary material for: Therapeutic Drug Monitoring of Orally Administered Letermovir Prophylaxis in Allogeneic Hematopoietic Stem Cell Transplant Recipients
Source: Antimicrob Agents Chemother. 2022 Jul 25;66(8):e00657-22. doi: 10.1128/aac.00657-22 (PMC9380536; doi:10.1128/aac.00657-22)

Supplemental material

Figure S1. Associations between letermovir plasma trough concentration, renal function (a), and liver function (b-c). C_{trough}: letermovir trough concentration. GFR: glomerular filtration rate, in mL/min/1.73 m2. AST: aspartate transaminase. ALT: alanine transaminase. Data are represented as boxplots in a log scale, with whiskers representing minimum and maximum values.

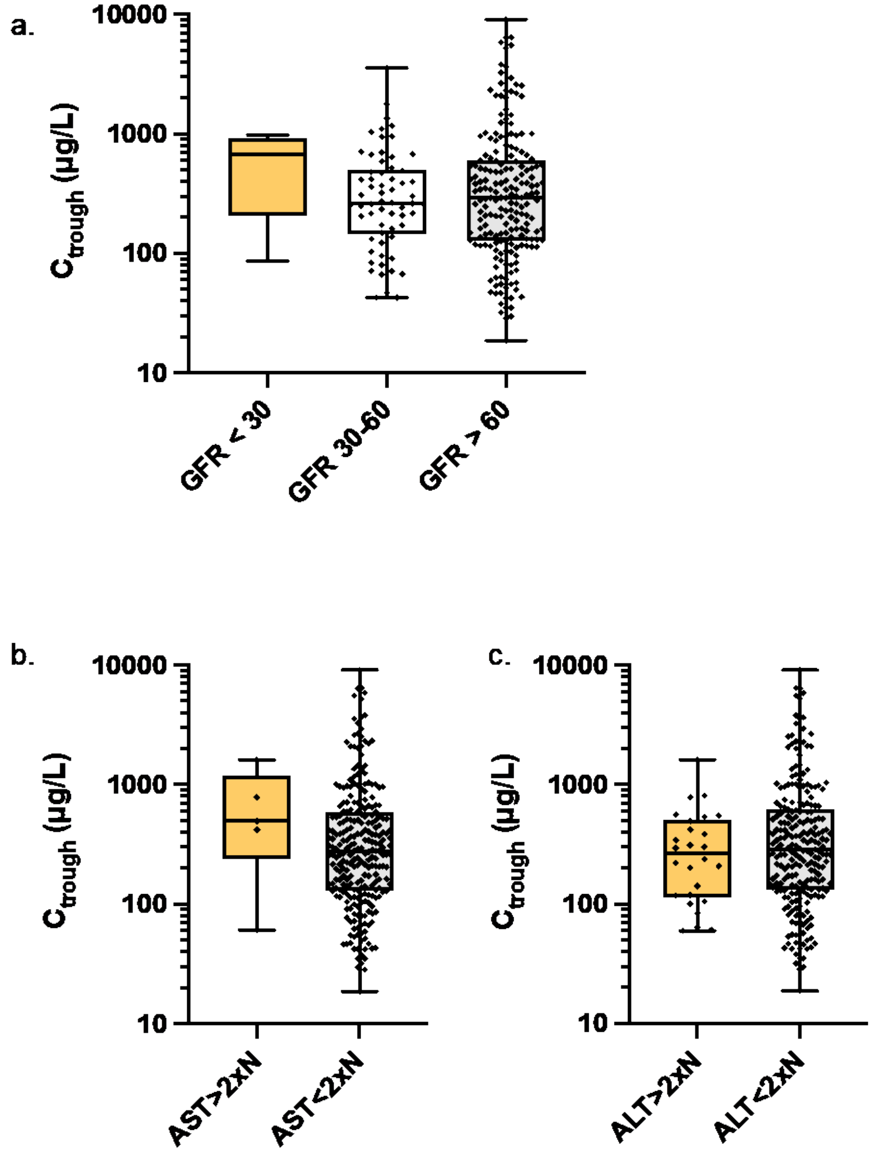

Supplement: Supplemental file 1 — Fig. S1. Download aac.00657-22-s0001.pdf, PDF file, 0.1 MB [file aac.00657-22-s0001.pdf]
